# Effect of hip abductors training on pelvic drop and knee valgus in runners with medial tibial stress syndrome: a randomized controlled trial

**DOI:** 10.1186/s13018-024-05139-3

**Published:** 2024-10-29

**Authors:** Shreen Ahmed Lashien, Ahmed Omar Abdelnaeem, Ebtessam Fawzy Gomaa

**Affiliations:** Department of Orthopedic Physical Therapy, Faculty of Physical Therapy, Cairo University, Cairo, Egypt

**Keywords:** Medial tibial stress syndrome, Hip, Exercise, Running

## Abstract

**Background:**

Medial tibial stress syndrome (MTSS) is a prevalent running-related injury that could impact athletic performance and quality of life. The purpose of the study was to investigate the effectiveness of functional hip abductor strength training on reducing contralateral pelvic drop angle (hip frontal plane projection angle), and dynamic knee valgus (knee frontal plane projection angle) in runners with medial tibial stress syndrome (MTSS).

**Methods:**

Forty male and female recreational runners were diagnosed with MTSS for at least one month. The age ranged from 25 to 35 years old, and the body mass index (BMI) ranged between (18.5 and 25 kg/m^2^) participated in this study. This was a single-blind, randomized controlled trial. Participants were randomized into two groups: a control group (Group A, n = 20) received a selected physical therapy exercise program, and an experimental group (Group B, n = 20) received the same program plus functional hip abductor strength training. Dynamic knee valgus (frontal plane projection angle) and contralateral pelvic drop angle were measured using 2D video and analyzed by Kinovea software at baseline and after 8 weeks. Mixed-effect multifactor analysis of variance (MANOVA) was conducted to compare within and between groups effects on FPPA and pelvic drop angle.

**Results:**

After 8 weeks, Group B exhibited a significantly decreased frontal plane projection and pelvic drop angles compared to Group A (*p* < 0.05). Group A also demonstrated a reduced pelvic drop angle, but to a lesser extent, and their frontal plane projection angle increased.

**Conclusions:**

This study demonstrates that 8 weeks of functional hip abductor strength training, combined with traditional physical therapy, effectively improves lower extremity kinematics in runners with MTSS by reducing dynamic knee valgus and contralateral pelvic drop. This targeted approach likely addresses underlying muscle weakness and movement dysfunction, offering hope for potentially reducing MTSS recurrence.

**Trial registration***:* clinicaltrials.gov. NO: NCT05637476. *Date:* December 1, 2022.

## Background

Medial tibial stress syndrome (MTSS), known as shin splints, is a prevalent running-related injury affecting the lower extremities [1, 2]. It accounts for 35 percent of sports injuries [3]. Characterized by diffuse pain and tenderness along the medial tibial border, MTSS can significantly impact athletic performance and quality of life [2].

Growing public interest in physical activity, particularly running, due to its accessibility and affordability, has driven the need for a deeper understanding of MTSS risk factors, causes, and optimal treatment strategies [4]. While activity typically exacerbates MTSS symptoms, pain often subsides with rest [5]. However, a notable challenge lies in the high recurrence rate of athletes returning to their sports [5]. Beyond pain, MTSS can lead to functional decline, potentially hindering daily activities and athletic participation [2].

The diagnosis of MTSS primarily relies on a combination of medical history, physical examination, and imaging studies such as X-rays, magnetic resonance imaging (MRI), or bone scans to rule out other potential causes of pain, like stress fractures [6].

The treatment targets eliminating pain, discomfort, and inflammation, identifying potential biomechanical impairments, and enhancing function on either a short-term or long-term basis, enabling individuals to generally return to their functional daily living activities and exercise routines [6]**.** Rest, ice, non-steroidal anti-inflammatory medications, compression, supportive insoles and return to exercise are common treatments for MTSS [6]. Also, a safe and efficient treatment option for chronic MTSS is low-energy shock wave therapy (SWT) [7]. The majority of athletes experience relief via conservative treatment, and surgical treatment such as fasciotomy of the deep posterior compartment and release of the periosteum is rarely needed, only in severe cases when nonsurgical treatment fails [6]**.**

The development of MTSS likely involves a combination of external and internal factors [6]. External factors include running on hard surfaces or using inappropriate footwear [8]. Internal factors such as a lack of flexibility training [8], gender [3], navicular drop, over-pronation of the foot [9], and weak hip abductors can contribute to poor dynamic limb alignment and control during weight-bearing activities [10]. This dynamic limb malalignment manifests as insufficient trunk, pelvic, hip, knee, or foot control in the frontal or transverse planes [11]. During lower limb activities, especially in a closed kinetic chain, the relationship from ASIS to the center of the patella and the tibial tubercle is continuously changing, as is the dynamic Q-angle [12]**.** The manner and magnitude to which the dynamic Q-angle changes during dynamic activities depend on individual biomechanical responses, the kinematic and kinetic nature of the movement patterns, and any associated pathology [13, 14]**.** Additionally, there is a critical correlation between the muscle activation of the involved muscles and the kinematics of the dynamic function of the lower limb, such as drop jump landing [15]**.** Therefore, hip abductors’ weakness and dynamic knee valgus predispose a higher risk of MTSS and are correlated with excessive foot pronation and ankle eversion, usually combined with tibial internal rotation, lateral patellar tracking, knee abduction, a greater femoral internal rotation, hip adduction/internal rotation, and contralateral pelvic drop, which in turn increase the medial ground reaction force and strain on the controlling muscles, such as the gluteal muscles, iliotibial band, and tensor fascia latae [16]**.**

Dynamic knee valgus, a highly reliable measure of this alignment assessed in the frontal plane, is a valuable tool for identifying this pathomechanical dysfunction [16].

The frontal and transverse planes of the dynamic quadriceps angle (Q-angle) appear promising biomechanical parameters, because they have clinical significance in diagnosing, avoiding, and treating various musculoskeletal disorders [16]. In addition, it can represent the real line of pull of the quadriceps during dynamic activities [16].

The concept that dysfunctional movement patterns can result from pathology and contribute to its development is gaining traction [17]. This perspective suggests that individuals with musculoskeletal pain syndromes often exhibit habitual movements and postures that play a pivotal role in the development of movement dysfunction [18].

In line with this concept and acknowledging the lack of gold standard treatment for MTSS, effective management hinges on a thorough assessment of the movement control deficiencies through clinical reasoning [18]. This paves the way for targeted retraining using a functional movement system approach [18].

Accordingly, this study aimed to investigate the effectiveness of functional hip abductor strength training in reducing contralateral pelvic drop angle and dynamic knee valgus in runners with MTSS. Our goal was to contribute to developing holistic and optimal exercise prescriptions for rehabilitation, prevention, and treatment, ultimately enabling runners with MTSS to improve their running performance and enhance their overall sports participation experience. We hypothesized that recreational runners diagnosed with MTSS receiving functional strength training for hip abductors in addition to a traditional selected physical therapy program would demonstrate greater improvement in contralateral pelvic drop angle and dynamic knee valgus in the frontal plane during a single leg/step-down test compared to those receiving only the traditional selected physical therapy program.

## Participants and methods

A randomized controlled trial was conducted at the orthopedic outpatient clinic of the Faculty of Physical Therapy, Cairo University, and Gezira Youth Center, Egypt, between January 2023 and January 2024. The study aimed to investigate the effect of functional hip abductor strength training on hip and knee kinematics in runners with MTSS. The research was conducted after obtaining ethical approval from the Faculty of Physical Therapy at Cairo University in Egypt (reference number: P.T.REC/012/004354). This was a single-blinded, randomized clinical trial. Participants were blinded by the treatment they received. However, the principal investigator (PI) was not blinded by the group assignment and was trained for the group allocation. Also, this trial has been registered at https://www.clinicaltrials.gov/ (registration number: NCT05637476).

### Participants

**Fig. 1:**
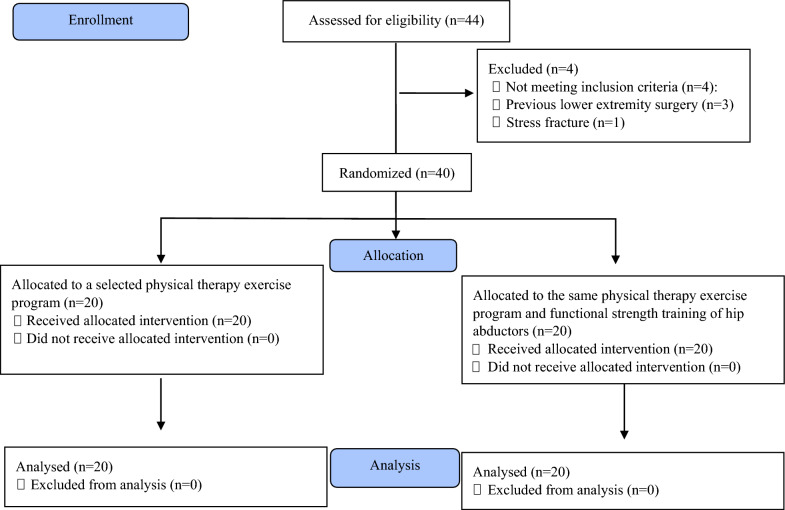
A flow chart illustrating the study's progression through its phases according to the CONSORT statements

Forty participants diagnosed with MTSS for at least one month were recruited (26 males, 14 females) and randomly assigned to two groups using an online randomization tool (http://www.randomization.com) (Fig. 1). Group A (Active Control, n = 20): received a selected physical therapy exercise program for 8 weeks. Group B (Experimental, n = 20): received the same physical therapy exercise program as Group A, plus additional functional hip abductor strength training for 8 weeks.

### Inclusion criteria

Participants were male and female recreational runners who ran for less than 5 h per week [19], with a referred diagnosis of MTSS for at least one month [9, 10]. The age range is (25–35 years), and the body mass index (BMI) range between (18.5 and 25 kg/m^2^) [8]. The measurements included the most affected limb in participants with bilateral MTSS. The diagnosis was confirmed clinically by increasing pain on passive dorsiflexion of the foot with great toe hyperextension and extreme passive plantar flexion [20]**.** The ankle’s range of motion might be decreased, limited to 20° dorsiflexion and 40–50° plantar flexion; there were no sensory, motor, or muscular abnormalities [20]**.**

In addition to tenderness of the posteromedial border of the tibia, particularly along the middle or distal third of its length [8]**.** We used a one-leg hop test to distinguish between MTSS and a stress fracture. A recreational runner with MTSS could hop at least 10 times on the affected leg, while a runner with a stress fracture couldn’t without severe pain [21].

### Exclusion criteria

Participants were excluded if they reported any conditions: recent, or old lower limb fractures lasting within the year before the test [22] or previous lower extremity surgery.

### Procedures

#### Anthropometric measure

Weight and height were measured using a calibrated weight scale to calculate the BMI (BMI = weight (kg)/height^2^ (m^2^)) [23].

#### *Assessment of the frontal plane projection angle (FPPA) dynamic knee valgus during the single-leg/step-down test pre and post treatment* (Fig. 2)

**Fig. 2:**
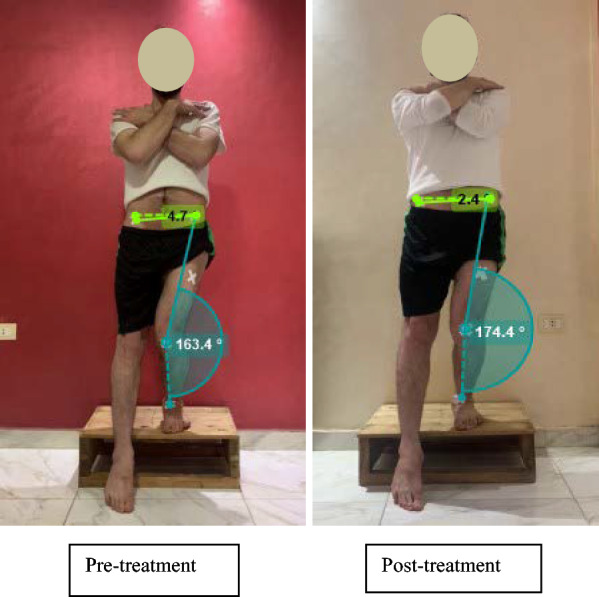
Assessment of the frontal plane projection angle (FPPA) dynamic knee valgus and the contralateral pelvic drop angle during the single-leg /step-down test: Pre and post treatment. Written ethical approval was obtained from participant for photographs

During a single-leg stance step-down test, the FPPA was assessed using 2D video recordings captured from a fixed tripod positioned two meters from the participant at the knee joint height [24]. The FPPA, as a measure of knee valgus, was calculated as the angle formed by two lines. Line 1: anterior superior iliac spine (ASIS) to the midpoint of the tibiofemoral joint. Line 2: midpoint of the tibiofemoral joint to the midpoint of the ankle malleoli of the stance (tested) leg, and measuring the resulting angle [25]. The measurement was then subtracted from a vertical line (180°) [26]. Our study investigated the lateral angle of the FPPA of the knee.

A positive FPPA (> 10°) indicated dynamic valgus; the knee marker was medial to the line from the anterior superior iliac spine to the midpoint of the ankle malleoli [25, 27, 28]. While a negative FPPA (> − 10°) indicated dynamic varus, the knee marker was lateral [25, 27, 28]. No change was defined as ≤ 10° in either direction [25, 27, 28].

Before assessment, reflective markers were placed on the participant’s (stance) lower extremity of the ASIS, the tibiofemoral joint midpoint (the midpoint between the femoral condyles), and the ankle malleoli midpoints for FPPA assessment. Similar markers were placed on both ASISs for contralateral pelvic drop assessment. Kinovea software (version 0.9.5) was used for the angular analysis [24]. Kinovea software analysis is a reliable and valid range of motion assessment technique [28] (Fig. 2).

Participants stood double-legged on a 20 cm step. They were instructed to squat on the tested (stance) leg and step down with the untested (swing) leg. This descent took 2 s, followed by 1 s of toe touching the ground, and a 2-s ascent back to the starting position, all monitored by a timer. The FPPA was measured at maximum knee flexion in the tested (stance) leg. In contrast, the untested (swing) leg reached its lowest point of the step-down test as the toe touched the ground [29],to a specific marked point 20 cm from the step in the anterior direction, which was aligned with the ASIS, the tibiofemoral joint midpoint (the midpoint between the femoral condyles), and the ankle malleoli midpoints for FPPA assessment. Three practice trials were performed with verbal instructions on the movement pattern without precise instructions on the position of the knee and hip [28], followed by three recorded trials for data analysis (using the average FPPA value) [29]. Hands were crossed in front of the chest to induce better neuromuscular activation of the lower limbs during the step-down test and balance by optimizing the position of the center of mass (COM) in the anterior–posterior direction [30].

#### *Assessment of the contralateral pelvic drop angle during the single leg /step-down test pre and post treatment* (Fig. 2)

The contralateral pelvic drop angle was defined as the inferior displacement of the hip marker on the non-stance (non-tested) leg, often resulting from gluteus medius weakness of the stance (tested) limb [24]. It was calculated as the angle between one line connecting the ASISs of both limbs and a second line drawn perpendicular to the stance limb ASIS. Then, the measurement was subtracted from 90° [31] (Fig. 2).

#### Treatment procedures

Both groups received a selected physical therapy exercise program for 8 weeks, consisting of three sets of 15 repetitions, 15 s rest between sets, three times per week, for the following exercises (Appendix 1):

1. Strength training for ankle dorsiflexors using a resistance band [32].
2. Eccentric calf exercise (calf raise) [32].
3. Balance and proprioceptive exercises on a balance board [32]. The balance exercises progressively increased: Each exercise was performed for 30 s per leg; legs were alternated during a 30-s rest between sets [33].
4. Static plantar flexor stretches (30 s duration hold, three sets, thirty seconds rest in between sets [20].

Group B (experimental): received the same physical therapy exercise program as Group A, plus additional functional hip abductor strength training. Exercises included (Appendix 1):

1. Pelvic drop [34].
2. Single-leg bridge [34].
3. Side-lying hip abduction with internal rotation [34].
4. Lateral step-up [34].
5. Standing hip abduction on stance or swing leg with extra resistance [34].

All exercises were progressed individually using various strengths of resistance bands and a repetition maximum (RM) method [33], specifically a ten repetition maximum 10-RM (the amount of weight that could be lifted and lowered 10 times through the entire ROM during exercise) [35]. Both groups ceased running activity for 3 weeks [3], followed by a gradual return to running at < 50% of their previous training volume [32].

Mixed-effect multifactor analysis of variance (MANOVA) was conducted to compare within and between groups effects on FPPA and pelvic drop angle. The significance level for all statistical tests was set at *p* < 0.05. All statistical analyses were performed using the Statistical Package for Social Sciences (SPSS) version 25 for Windows (IBM SPSS, Chicago, IL, USA).

## Results

### Subject characteristics

There was no significant difference between groups in age, BMI, sex and affected side distribution (*p* > 0.05) (Table 1).

**Table 1:** Basic characteristics of participants

	Group A	Group B			
	Mean ± SD	Mean ± SD	MD	t- value	p-value
Age (years)	26.20 ± 1.54	27.15 ± 2.46	-0.95	-1.46	0.15
BMI (kg/m^2^)	21.85 ± 1.79	21.68 ± 1.61	0.17	0.31	0.76
*Sex, n (%)*					
Females	6 (30%)	8 (40%)		(χ^2^ = 0.44)	0.51
Males	14 (70%)	12 (60%)			
*Affected leg, n (%)*					
Right	7 (35%)	5 (25%)		(χ^2^ = 0.47)	0.49
Left	13 (65%)	15 (75%)			

SD, standard deviation; MD, mean difference; χ^2^, Chi squared value p-value, level of significance

### Effect of treatment on FPPA and contralateral pelvic drop angle

Mixed MANOVA revealed a significant interaction effect between treatment and time (F = 35.43, *p* = 0.001, partial eta squared = 0.66). There was a significant main effect of time (F = 33.71, *p* = 0.001, partial eta squared = 0.65). The main effect of treatment was significant (F = 5.44, *p* = 0.008, partial eta squared = 0.23) (Table 2).

**Table 2:** Mixed MANOVA for the interaction effect between treatment and time

Mixed MANOVA			
Interaction effect (treatment * time)			
*F* = *35.43*	*p* = *0.001*	*S*	*η*^*2*^ = *0.66*
*Effect of time*			
*F* = *33.71*	*p* = *0.001*	*S*	*η*^*2*^ = *0.65*
*Effect of treatment*			
*F* = *5.44*	*p* = *0.008*	*S*	*η*^*2*^ = *0.23*

F value, Mixed MANOVA F value; p-value, Probability value; S, Significant; η^2^, partial eta squared

### Within-group comparison

**FPPA**: Group B (intervention) demonstrated a significant decrease in FPPA (indicating improved knee alignment) after treatment compared to baseline (*P* < 0.001). Conversely, Group A (control) exhibited a significant increase in FPPA (worsening knee alignment) post-treatment compared to baseline (*p* < 0.001).

**Pelvic Drop Angle**: Both groups (A and B) showed a significant decrease in contralateral pelvic drop angle (improved pelvic stability) post-treatment compared to baseline (*p* < 0.01).

The percent change of FPPA and contralateral pelvic drop angle in group A was 28.41 and 19.42%, respectively, and that of group B was 42.05 and 49.85%, respectively (Table 3).

**Table 3:** Mean FPPA and pelvic drop angle pre and post-treatment of groups A and B

	Group A	Group B		
	Mean ± SD	Mean ± SD	MD (95% CI)	p-value
*FPPA (degrees)*				
Pre-treatment	9.82 ± 3.14	10.63 ± 2.79	− 0.81 (− 2.71: 1.09)	0.39
Post-treatment	12.61 ± 3.51	6.16 ± 2.80	6.45 (4.39: 8.53)	0.001
MD (95% CI)	− 2.79 (− 4.17: − 1.42)	4.47 (3.10: 5.85)		
% of change	28.41	42.05		
	*p* = *0.001*	*p* = *0.001*		
*Pelvic drop angle (degrees)*				
Pre-treatment	6.18 ± 2.92	6.86 ± 2.09	− 0.68 (− 2.31: 0.94)	0.40
Post-treatment	4.98 ± 2.51	3.44 ± 1.98	1.54 (0.09: 2.99)	0.03
MD (95% CI)	1.2 (0.38: 2.01)	3.42 (2.61: 4.24)		
% of change	19.42	49.85		
	*p* = *0.005*	*p* = *0.001*		

SD, Standard deviation; MD, Mean difference; *p*-value, Probability value

### Between-group comparison

**Fig. 3:**
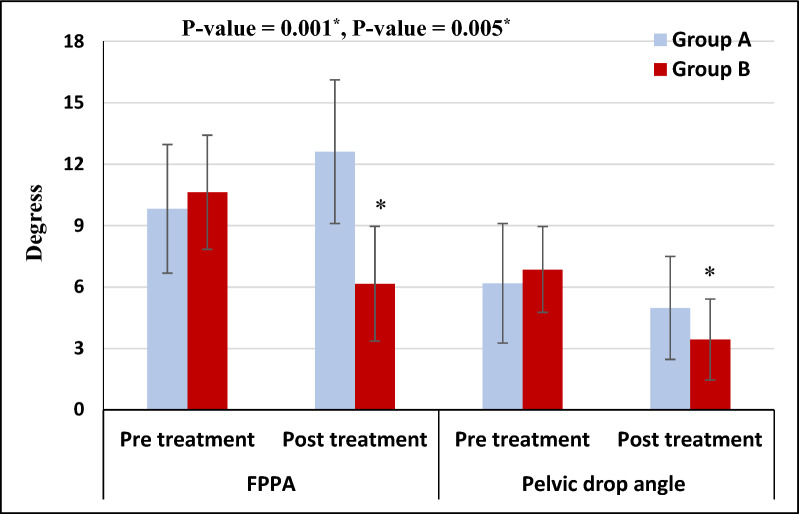
Mean FPPA and contralateral pelvic drop angle pre and post-treatment of groups A and B

Post-treatment, Group B demonstrated a significantly greater decrease in both FPPA, and contralateral pelvic drop angle compared to Group A (*p* < 0.05) (Table 3). This finding suggests that the functional hip abductor strength training program in Group B improved knee alignment and pelvic stability compared to the standard physical therapy program in Group A (Fig. 3).

## Discussion

This study aimed to investigate the effectiveness of functional hip abductor strength training in reducing contralateral pelvic drop angle and dynamic knee valgus in recreational runners (those running less than 5 h weekly) diagnosed with medial tibial stress syndrome (MTSS) [19].The primary hypothesis was that recreational runners diagnosed with MTSS receiving functional strength training for hip abductors in addition to a traditionally selected physical therapy program would demonstrate better improvement in contralateral pelvic drop angle and dynamic knee valgus in the frontal plane during a single leg/step-down test compared to those receiving only the traditional selected physical therapy program.

The results demonstrate a significant decrease in FPPA and pelvic drop angle in group B compared to group A post-treatment. Group B received functional strength training for hip abductors in addition to the traditional selected physical therapy program, while Group A received only the traditional selected physical therapy program. Notably, group A also showed a decrease in pelvic drop angle, but less than group B. The significant decrease in dynamic knee valgus in group B compared to the significant increase in group A, while part of exercises for both groups was the same, suggests that, under normal circumstances, a balance between fatigue damage accumulation and remodeling activity maintains bone integrity [36]. However, high stress levels are linked to accelerated remodeling and bone damage due to fatigue [36]. Moreover, weak muscles are unable to absorb the shock, leading to transmitting the ground reaction force to the bone-muscle interface [36]. Thus, the mechanics of bone and the muscular activity in generating and attenuating stress are crucial factors that influence the level of bone remodeling activity [36].

This means any exercise-induced lower extremity pain syndromes may include soft tissue injuries to muscles, tendons, and ligaments and disturbances in bone remodeling leading to a stress reaction in bone, such as MTSS, which may proceed to a stress fracture [36]. Considering both groups with MTSS ceased running activity for 3 weeks [3] while doing the exercise program according to their group, followed by a gradual return to running at < 50% of their previous training volume [32], with continuity practicing their exercise program.

Accordingly, the significant decrease in dynamic knee valgus (FPPA) in group B represents the critical value of hip abductors' added functional strength training, accurate clinical reasoning and proper lower limb biomechanical assessment, taking into consideration the hip abductors’ weakness as the main reason for the dynamic knee valgus and contralateral pelvic drop [37]**.** The primary role of the hip abductors’ is to maintain pelvic level during single-leg stance, as in walking and running [37]**.** Therefore, we prescribed a holistic rehabilitation exercise program that treats the leading causes and the lower limb as one unit, not only the symptoms.

This, in turn, means that the increase in Dynamic knee valgus (FPPA) observed in group A reinforces the concept that adaptations can occur proximally to distally and vice versa; however, in lower limb injuries, distal to proximal adaptations are more common [38]. This highlights the interconnected nature of the lower extremity kinetic chain. Therefore, during gait, movements like foot pronation can lead to tibial internal rotation, which can cause hip internal rotation and knee valgus [38]. Any alteration in timing or force generation within this chain can induce pathology or poor performance at another level [39]. This is particularly relevant for high-impact activities like running, where poor biomechanical alignment increases the vulnerability of the lower limb, exposing the athlete to a higher risk of injury (tibial bone stress injury, patellofemoral pain, and plantar fasciitis) due to the greater loads and ground reaction forces associated with such activities [40]. On the other hand, the observed decrease in pelvic drop angle in both groups suggests that this parameter may be a sensitive indicator of treatment response. However, the more pronounced reduction in Group B emphasizes the importance of targeted hip muscle strengthening in addressing this biomechanical factor [38]. Additionally, sensorimotor training effectively enhances static balance but not pelvic stability or dynamic balance [41].

Our findings align with Hadadnezhad and Sheikhi, who reported that individualized, feedback-based hip muscle resistance training improved knee valgus, contralateral pelvic drop, and lateral trunk flexion in female volleyball players with dynamic valgus during landing within 6 weeks [31]. Similarly, researchers suggested that runners who develop MTSS exhibit weaker hip abductors and a higher contralateral pelvic drop [32]. To evaluate these biomechanical parameters, our current study used 2D video analysis. This method offers a reliable, accurate, and easily accessible tool for assessing running mechanics, including contralateral pelvic drop angle and unilateral dynamic knee valgus from an anterior view during a step-down test, which aligns with the established utility of functional movement analysis for lower extremity pain syndromes, including step-down tests [38]. However, it is essential to acknowledge that 2D analysis has limitations, as it does not capture movement in all planes.

Our findings’ anatomical explanation lies in the femur’s obliquity anatomical (longitudinal) axis [42]. This natural angulation creates a physiological valgus of up to five degrees from the vertical line [42]. In contrast, the lower limb's mechanical axis (weight-bearing line) travels from the center of the hip joint to the center of the ankle joint, passing through the midpoint of the tibiofemoral joint [43]. This mechanical axis represents the ground reaction force as it transmits up through the lower extremity [42]. In a neutrally aligned limb, the weight-bearing forces distribute equally between the medial and lateral condyles of the knee joint [43].

However, during single-leg stance (the stance phase of gait and running), the mechanical axis shifts toward the medial part of the knee joint to accommodate the narrower base of support below the center of mass [42]. This medial shift amplifies the compressive stress on the medial compartment and the tensile stress on the lateral compartment [44]. Thus, any altered or abnormal anatomical alignment increases the lateral compressive force as the weight-bearing line shifts onto the lateral compartment of the knee (genu valgus), increasing the lateral compressive force [42]. Conversely, a medial shift of the weight-bearing line (genu varum) increases the medial compressive force [42]. Furthermore, recent studies support that MTSS arises from an overload of the medial tibia [45]. This vulnerability is likely due to the medial surface being subcutaneous and having the thinnest bone section (junction between the upper two-thirds and lower third of the shaft) with the poorest blood supply [46].

As per our literature search, running mechanics differ from walking, requiring greater balance, muscle strength, and joint range of motion [47]. Notably, running lacks the double support phase present during the walking gait cycle [47]. Instead, each running cycle has a unique double floating phase at the beginning of 15% and at the end of 15% of the swing phase when both feet are off the ground [47]. The running cycle includes two phases: the stance phase accounts for 40% of the running cycle, while the swing phase accounts for 60% [47]. During single-limb support in running, the hip abductors, particularly the gluteus medius, play a crucial role in the frontal and transverse planes [47]. In the absorption phase, the gluteus medius contracts eccentrically to control contralateral pelvis drop, hip adduction, internal rotation, and knee abduction (dynamic knee valgus), which occurs as the ground reaction force falls medial to the hip and the hip abductor moment is less than the external adduction moment due to gravity and acceleration load [47]. While in the propulsion phase, the gluteus medius contracts concentrically to abduct the hip and generate power [47]. Weakness in the gluteus medius can lead to excessive hip adduction and contralateral pelvic drop [38].

Consequently, several recent studies have highlighted the insufficient evidence for treatment guidelines and the badly needed investigation of the efficacy of functional strength training of hip abductors for vulnerable athletes with poor dynamic lower limb alignment at higher risk of sports injuries [31]. Therefore, we advocated five functional strength training exercises for hip abductors as a promising approach to conventional treatment exercises for MTSS.

Considering the application of the overload principle, which guides us to use a resistance load greater than the metabolic capacity of the muscle to enhance its performance [48]. To achieve sufficient strength improvements, functional training should target the gluteus medius with an intensity of at least 60% of its maximum voluntary contraction (MVC) [49]. This explains the importance of these five exercises in our functional training, as according to the systematic review by Moore et al., these are the best exercises to provide at least 60% of the maximum contraction of the gluteus medius in particular [34]. Integrating functional strength exercises as an essential priority in rehabilitation enhances the neuromuscular system’s ability to generate the proper amount of force during functional activities in a smooth and coordinated manner [33].

Consequently, the training program adhered to periodized training concepts, which emphasize the interaction between stability and active movement, muscle strength requiring motor capabilities before the overloading principle, and task-specific movement patterns with resistance exercise [33].

Taking into consideration, after identifying the baseline RM for a particular training-induced adaptation in strength, a percentage of an exercise program ranges from 60 to 70% of the baseline RM for healthy, untrained adults [50], and > 80% of the baseline RM for highly trained athletes is needed [33].

Finally, our program duration of 8 weeks aligns with research by Moore et al., who observed initial and rapid gains in muscular force-generating capacity as an increase in electromyographic activity occurs within this timeframe of resistance training [33]. This initial gain is primarily attributed to neural adaptations with minimal muscle fiber hypertrophy, while increased muscle fiber hypertrophy and vascularization typically occur within six to twelve weeks of training [51].

It is also worth recognizing that female athletes are more susceptible to sports injuries as they have greater dynamic knee mal-alignment due to wider pelvis diameter, greater ligament laxity, a smaller acetabulum, and a wider subpubic angle [24].

## Limitations and future directions

Overall, the 2D motion analysis only captured movement in the frontal plane, neglecting potential contributions from other planes. Future research should incorporate 3D motion analysis to assess running mechanics comprehensively. Additionally, our study focused on recreational runners. Investigating the effects of hip abductor training on long-distance runners with different training volumes and risk factors for MTSS is warranted. Runners with higher mileage and greater training intensity may experience different stress levels on the lower limb and respond differently to intervention.

Furthermore, exploring the potential benefits of integrating additional therapies like hydrotherapy, shockwave therapy, and kinesio-taping in conjunction with the standard treatment could optimize MTSS management. Research in this area could help develop a more comprehensive treatment approach for MTSS, potentially improving patient outcomes. Investigating the development of footwear specifically designed for novice runners based on our findings on injury risk factors could also be a valuable area for future research. Footwear design that addresses biomechanical imbalances and provides better support could play a role in preventing MTSS development.

The results highlight the importance of incorporating hip abductor strengthening exercises into rehabilitation programs for MTSS, particularly in athletes with dynamic knee valgus and contralateral pelvic drop angle. Additionally, the study emphasizes the potential utility of 2D motion analysis as a screening tool for identifying injury risk and monitoring treatment progress. Implementing such tools in clinical practice can allow for earlier intervention and potentially improve long-term patient outcomes.

Developing a mobile application incorporating screening tools based on our findings could be a valuable resource for clinicians, enhancing clinical reasoning and facilitating the evaluation of treatment effects. This mobile app could provide clinicians with an easy-to-use tool for identifying runners at risk of MTSS and monitoring their progress during rehabilitation. The app could also offer educational resources for runners on injury prevention strategies and proper running form.

## Conclusion

Eight weeks of combined functional hip abductors strength training and the traditionally selected physical therapy exercise program significantly improved reductions in contralateral pelvic drop angle and dynamic knee valgus (knee frontal plane projection angle) in recreational runners with medial tibial stress syndrome, compared to the traditionally selected physical therapy exercise program alone.

## Data Availability

All data supporting the findings of this study are available within the paper.

## Appendix 1

Written ethical approval was obtained from participant for photographs.

## Abbreviations

MTSS: Medial tibial stress syndrome
FPPA: Frontal plane projection angle
Q-angle: Quadriceps angle
MRI: Magnetic resonance imaging
SWT: Low-energy shock wave therapy
PI: Principal investigator
CONSORT: Consolidated standards of reporting trials
ASIS: Anterior superior iliac spine
BMI: Body mass index
COM: Center of mass
RM: Repetition maximum
MANOVA: Multivariate analysis of variance
MVC: Maximum voluntary contraction
2D motion: Two-dimensional motion
3D motion: Three- dimensional motion
